# Population pharmacokinetics of pyrazinamide and ethambutol in children with tuberculosis with or without HIV

**DOI:** 10.1128/aac.00909-25

**Published:** 2026-03-02

**Authors:** Nicole F. Maranchick, Charles Martyn-Dickens, Anthony Enimil, Hongmei Yang, Aikins Kofi Amissah, Albert Dompreh, Dennis Bosomtwe, Eugenia Sly-Moore, Theresah Opoku, Augustina Frimpong Appiah, Priscilla Asiedu, Sampson Antwi, Marc H. Scheetz, Charles A. Peloquin, Awewura Kwara

**Affiliations:** 1 Infectious Disease Pharmacokinetics Laboratory, College of Pharmacy and Emerging Pathogens Institute, University of Florida 3463, Gainesville, Florida, USA; 2 Directorate of Child Health, Komfo Anokye Teaching Hospital 259295, Kumasi, Ghana; 3 Department of Child Health, School of Medical Sciences, Kwame Nkrumah University of Science and Technology 98763, Kumasi, Ghana; 4 Department of Biostatistics and Computational Biology, University of Rochester School of Medicine and Dentistry 12299, Rochester, New York, USA; 5 Department of Clinical Microbiology, Komfo Anokye Teaching Hospital 259295, Kumasi, Ghana; 6 Department of Medical Diagnostics, Kwame Nkrumah University of Science and Technology 98763, Kumasi, Ghana; 7 Department of Pharmacy, Komfo Anokye Teaching Hospital 259295, Kumasi, Ghana; 8 Department of Pharmacy Practice, College of Pharmacy, Midwestern University 69281, Downers Grove, Illinois, USA; 9 Pharmacometrics Center of Excellence, Midwestern University 69281, Downers Grove, Illinois, USA; 10 Department of Medicine, University of Florida College of Medicine 12233, Gainesville, Florida, USA; City St George's, University of London, London, United Kingdom

**Keywords:** ethambutol, pyrazinamide, anti-tuberculosis drugs, population pharmacokinetics, pediatrics

## Abstract

Tuberculosis (TB) is a major cause of morbidity and mortality in children globally. This study developed models to describe population pharmacokinetics (PK) of pyrazinamide (PZA) and ethambutol (EMB) in children with TB with or without human immunodeficiency virus (HIV) coinfection. Ghanaian children with TB with or without HIV coinfection receiving first-line antituberculosis therapy for at least 4 weeks had blood samples collected at time 0 (pre-dose), 1-, 2-, 4-, 8-, and 12-h post-dose. PZA and EMB concentrations were quantified using liquid chromatography tandem mass spectrometry. Nonlinear mixed-effects models were applied to describe the population PK using Monolix2024R1. Maximum concentrations (*C*_max_) and 24-h area under the time concentration curve (AUC_0–24_) were compared to published values in adults. A total of 85 children (41 TB, 44 TB/HIV) were included. The median (range) age was 5 years (0.3–14.5), and 61.2% were male. Median (range) doses for PZA and EMB were 31.6 (21.4–49.7) and 21.4 mg/kg (14.3–34.2), respectively. PZA was best described using a one-compartment model and EMB by a two-compartment model. Allometric scaling improved both model fits. Children with TB/HIV coinfection had approximately 18.5% faster PZA clearance and 25% faster EMB clearance. Optimized dosing to achieve adult-equivalent exposures required higher-than-currently recommended doses, particularly among children in the lowest weight bands and those with HIV. The population PK of PZA and EMB was well described by the final models, but the higher-than-currently recommended doses needed to achieve adult-equivalent exposures raise concerns regarding risks for drug-associated toxicities and will require further evaluation.

## INTRODUCTION

Tuberculosis (TB) is the leading infectious cause of mortality globally and a major contributor to morbidity and mortality in children (1). In 2024, an estimated 1.2 million children and young adolescents developed TB, representing roughly 11% of all reported cases (1). During the same period, mortality was approximately 174,000 among HIV-negative children and young adolescents and about 2,300 among their HIV-positive counterparts (1). For those living with HIV, there is approximately a 20–30-fold increased relative risk of developing TB disease from a latent state (2, 3), and children living with HIV are at increased risk for more severe forms of TB, such as meningitis or disseminated disease (4). Additionally, the risk of progression from infection to severe disease occurs more frequently in the first year post-exposure in children less than 2 years of age, often without prior symptoms (5). Mortality is high in children with TB/HIV coinfection, even when treatment is initiated, and mortality further increases with therapy delays or a lack of antiretroviral therapy (ART) (6).

The current World Health Organization (WHO) recommendations suggest first-line treatment with HRZE (isoniazid, rifampin, pyrazinamide, ethambutol) for children with drug-susceptible TB (7). In the 2 months of the intensive phase of therapy, participants receive all four drugs, followed by 4 months of rifampin and isoniazid. Pyrazinamide (PZA) is a key sterilizing agent and works well against TB bacilli in acidified portions of lesions, ultimately shortening treatment duration (8–10). Ethambutol (EMB) has moderate early bactericidal activity against TB bacilli (11). However, its main role is to protect rifampin in the event of isoniazid resistance (10, 11). Previously, anti-TB dose (mg/kg) recommendations in children were equivalent to adults, leading to suboptimal exposure (12, 13). To improve drug exposure, higher doses were recommended by the WHO in 2010 (14), and updated fixed-dose combinations (FDCs) have since been made available in accordance with these recommendations (15). However, even with new FDCs, drug exposure may still be suboptimal, and higher doses may be needed, especially in settings with a high HIV prevalence (16–19).

Given the importance of PZA and EMB in the first 2 months of treatment and the potential for suboptimal drug exposure, this study aimed to develop population pharmacokinetic (PK) models to describe PZA and EMB plasma profiles in children with TB or TB/HIV and to simulate target attainment with various doses across weight bands.

## RESULTS

A total of 85 participants contributed 519 samples (Table 1). During the study period, 92 participants were enrolled, but six did not complete PK sampling (four were lost to follow-up; one had a spinal tumor; and one was too sick for sampling). One patient had 0 mg/L concentrations for all EMB and PZA samples and was removed from the analysis. Fifty-two participants (61.2%) were male, and the median (range) age was 5 years (0.3–14.5). Forty-two (49.4%) participants were less than 5 years old, and 16 (18.8%) were less than 2 years old. The median (range) weight was 16 kg (4–60). Forty-one (48.2%) participants had TB, and 44 (51.8%) had TB/HIV. Of the participants with TB/HIV, 29 (65.9%) received efavirenz-based ART.

**TABLE 1:** Baseline demographics and clinical characteristics of participants, *n* = 85^*d*^

Characteristic	Number (%) or median (range), all (*n* = 85)	Number (%) or median (range), TB only (*n* = 41)	Number (%) or median (range), TB/HIV (*n* = 44)	*P*-value^*a*^
Age, years	5.0 (0.3–14.5)	4.1 (0.3–14.5)	5.0 (0.5–13)	0.51
<5	42 (49.4%)	21 (51.2%)	21 (47.7%)	
<2	16 (18.8%)	9 (22%)	7 (15.9%)	
Weight, kg	16 (4–60)	16 (5–60)	16.1 (4–31)	0.92
Fat-free mass, kg	15.2 (3.3–45.3)	14.3 (4.5–45.3)	15.5 (3.3–29.5)	0.86
Sex, male	52 (61.2%)	21 (51.2%)	31 (70.5%)	0.08
Weight band, kg				0.04^*b*^
4 to <8	11 (12.9%)	4 (9.8%)	7 (15.9%)	
8 to <12	12 (14.1%)	10 (24.4%)	2 (4.5%)	
12 to <16	17 (20%)	6 (14.6%)	11 (25%)	
16 to <25	31 (36.5%)	12 (29.3%)	19 (43.2%)	
≥25	14 (16.5%)	9 (22.0%)	5 (11.4%)	
Malnutrition^*c*^	24 (28.2%)	9 (22.0%)	15 (34.1%)	0.24
Dose (mg/kg)				
Pyrazinamide	31.6 (21.4–49.7)	31.6 (21.4–38.1)	31.8 (21.4–49.7)	0.53
Ethambutol	21.4 (14.3–34.2)	21.1 (14.3–25.2)	21.4 (15.4–34.2)	0.43
Serum creatinine (µmol/L), *n* = 82	24 (10–139)	27.5 (11–139)	22 (10–137)	0.03^*b*^
ALT (IU/L), *n* = 82	16 (7–160)	16 (7–34)	17 (7–160)	0.43
AST (IU/L), *n* = 82	37 (19–228)	35 (19–67)	43.5 (24–228)	0.02^*b*^
Total bilirubin (µmol/L), *n* = 82	6 (3–24)	5 (4–12)	6 (3–24)	0.34
ART-based regimen				
ABC	–^*e*^	–	7 (15.9%)	
DTG	–	–	4 (9.1%)	
EFV	–	–	29 (65.9%)	
LPV	–	–	4 (9.1%)	

^*a*^ *P*-value compares TB vs TB/HIV groups.

^*b*^ Designates statistically significant (*P *< 0.05).

^*c*^ Malnutrition defined as body mass index for age Z score < −2 standard deviations.

^*d*^ AST, aspartate aminotransferase; ALT, alanine aminotransferase; ART, antiretroviral therapy; ABC, abacavir; DTG, dolutegravir; EFV, efavirenz; LPV, lopinavir (co-administered with ritonavir).

^*e*^ –, no data is available.

### Pyrazinamide

**Fig 1:**
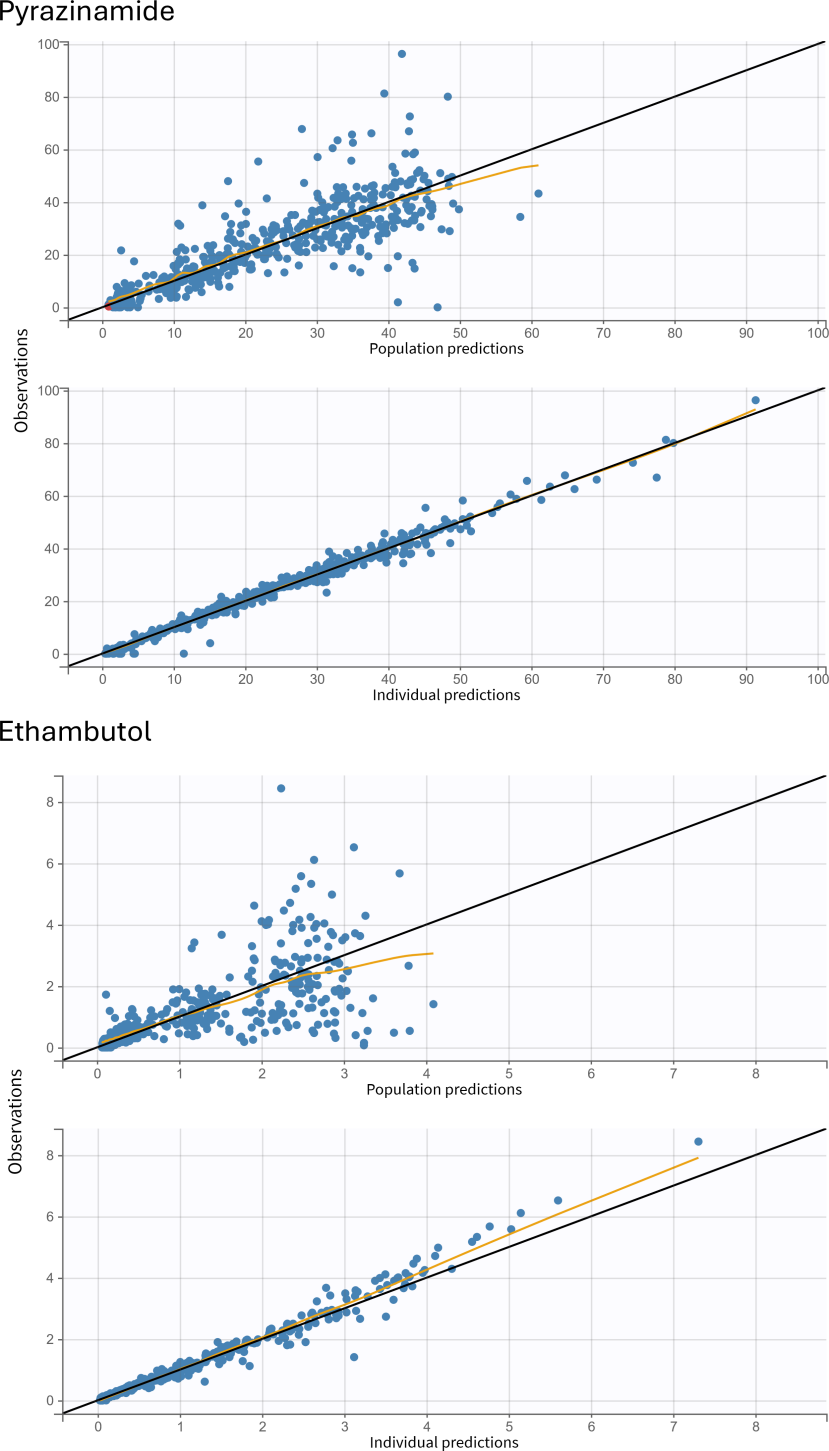
Goodness-of-fit plots for the pyrazinamide and ethambutol final population pharmacokinetic models. The top panels represent the population predicted concentrations versus observed concentrations, and the bottom panels represent the individual predicted concentrations versus observed concentrations. The yellow line represents the spline. In the pyrazinamide panel, samples below the limit of quantification are represented by red dots.

**Fig 2:**
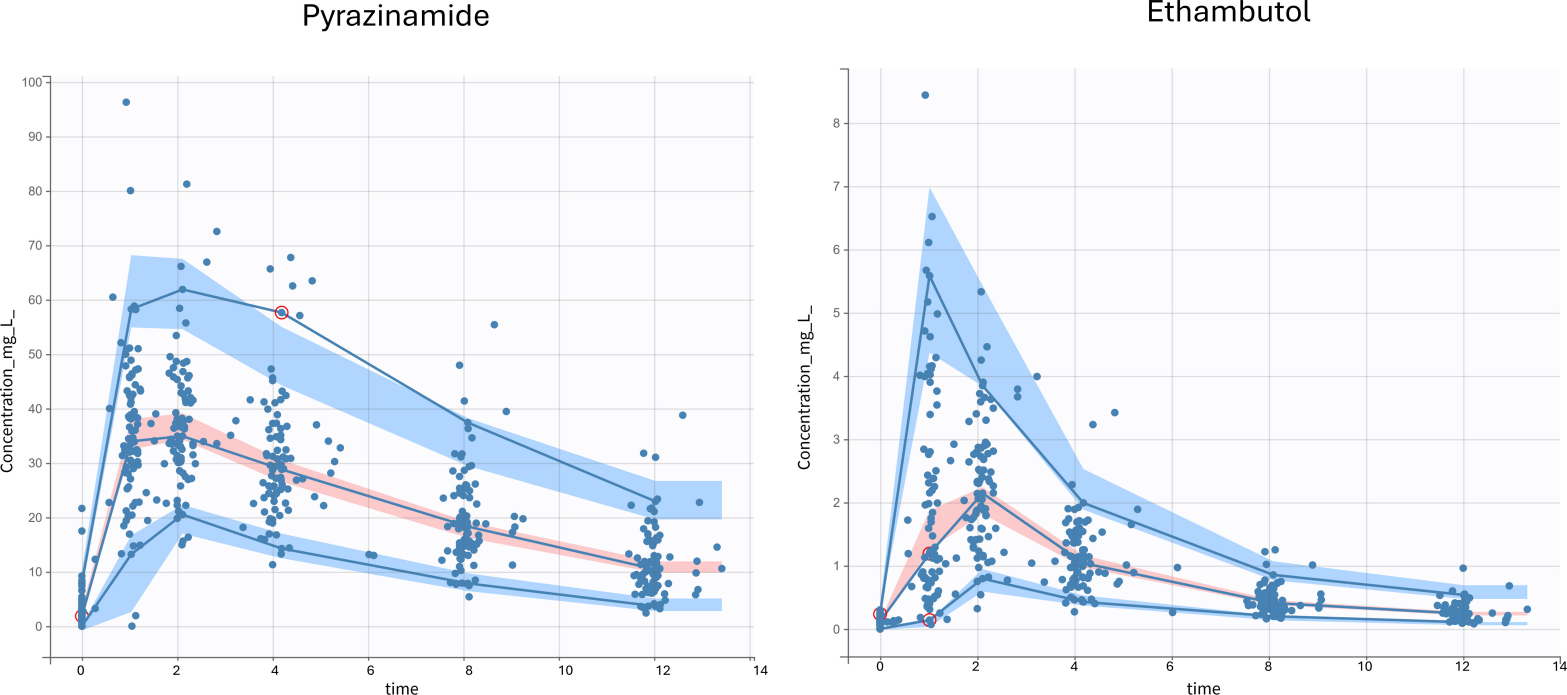
Visual predictive check plots for each drug concentration versus time (1,000 Monte Carlo simulations). The solid blue lines represent the 5th, 50th, and 95th percentiles of observations. The shaded regions represent the 90% confidence intervals around the 5th (blue), 50th (pink), and 95th (blue) percentiles of simulated data. The blue dots represent observed concentrations.

A total of 509 samples from 85 participants were included in the final model. The median (range) dose administered was 31.6 mg/kg (21.4–49.7). Sixteen samples were below the limit of quantification (BLQ); 14 were 0 mg/L, and two samples had drug detected, which were interval-censored. The population PK of PZA was described by a one-compartment model, first-order absorption with tlag, and linear elimination (Table 2). Weight was allometrically scaled using estimated coefficients on Cl/F (Cl/F*(Weight/15)^0.7) and V/F (V/F*(Weight/15)^0.79), which reduced the objective function value (OFV) by 108 and improved diagnostic plots. Children with TB/HIV had clearance that was 18.5% faster than children with TB. The goodness-of-fit plots (Fig. 1) and visual predictive checks (VPCs) (Fig. 2) indicated that the population PK of PZA in children was sufficiently described by the final model. Incorporating a maturation function did not improve model fit, perhaps because a small proportion of patients were <2 years old (18.8%) and even fewer (7.1%) younger than 1 year old. Additionally, the influence of inter-occasion variability (IOV) on PK parameters and HIV medications was assessed, but effects did not enhance model fit.

**TABLE 2:** Final population pharmacokinetic model parameter estimates for pyrazinamide and ethambutol^*a*^

Drug	Parameter	Estimate (RSE, %)	IIV (% CV)
Pyrazinamide	tlag (h)	0.26 (46.86)	0.75 (87.16)
	Ka (h^−1^)	3.76 (20.79)	0.62 (68.71)
	V/F (L)	11.30 (2.87)	0.23 (23.4)
	Exponent, BW on V/F	0.79 (6.64)	–^*b*^
	Cl/F (L/h)	1.27 (4.46)	0.32 (32.6)
	Exponent, HIV+ on Cl/F	0.17 (31.29)	–
	Exponent, BW on Cl/F	0.70 (9.74)	–
	Residual variability		
	a	1.06 (11.62)	–
	b	0.06 (13.33)	–
Ethambutol	tlag (h)	0.67 (7.04)	0.35 (36.35)
	Ka (h^−1^)	3.83 (22.97)	1.18 (172.61)
	Cl/F (L/h)	23.2 (4.4)	0.32 (33.26)
	Exponent, HIV+ on Cl/F	0.22 (21.03)	–
	Exponent, BW on Cl/F	0.70 (10.31)	–
	V1/F (L)	95.16 (6.49)	0.51 (54.63)
	Exponent, BW on V1/F	0.62 (19.34)	–
	Q/F (L/h)	11.25 (6.69)	0.34 (35.23)
	V2/F (L)	162.41 (16.45)	0.81 (96.75)
	Residual variability		
	a	0.03 (9.99)	–
	b	0.17 (7.08)	–

^*a*^ RSE; relative standard error; IIV, interindividual variability (reported as standard deviation of the random effects); CV, coefficient of variability; BW, body weight; HIV+, human immunodeficiency virus positive.

^*b*^ –, no data is available.

**Fig 3:**
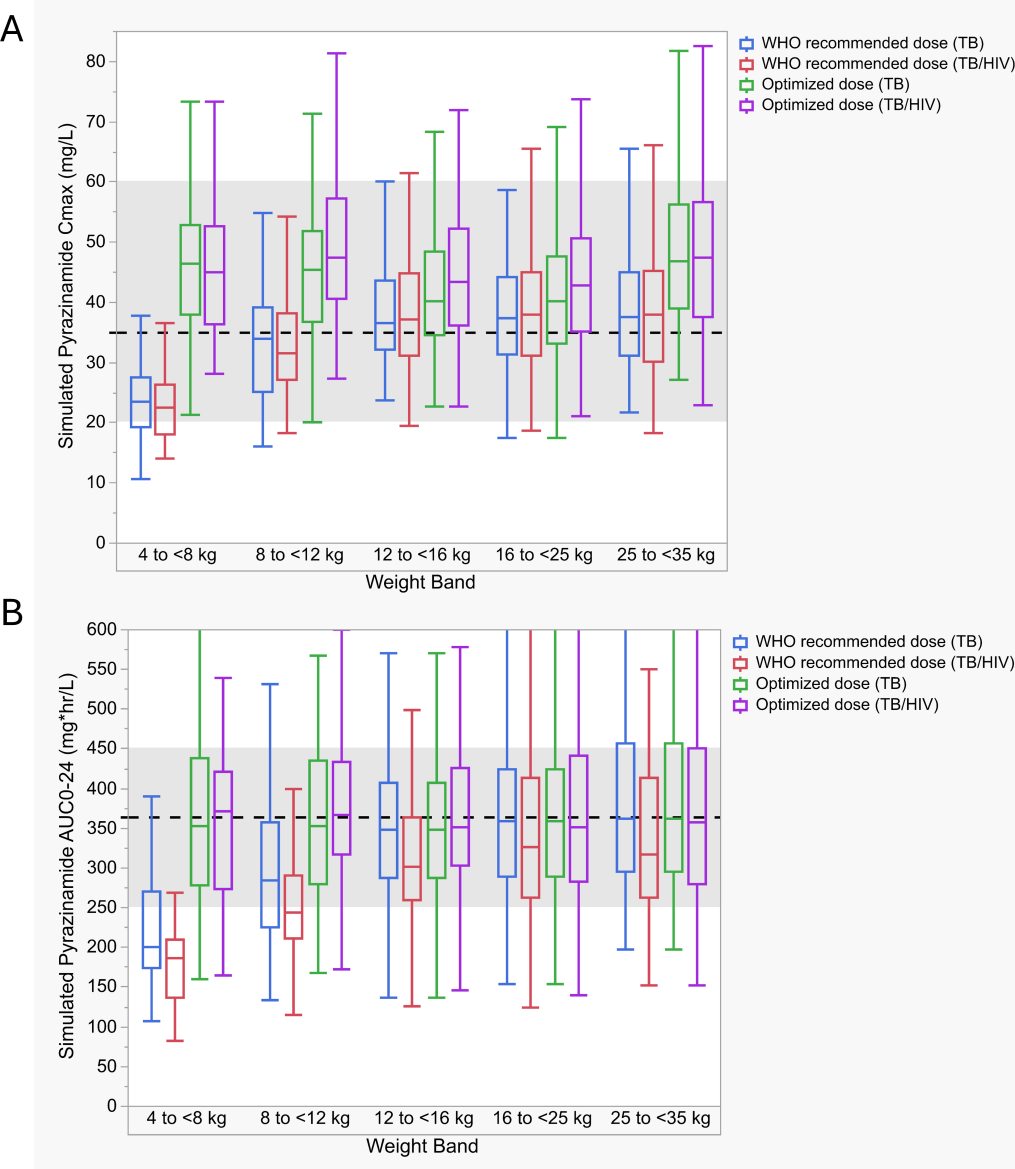
Simulated WHO-recommended and optimized pyrazinamide *C*_max_ and AUC_0–24_ values stratified by weight band and HIV status. Boxplots of simulated steady-state pyrazinamide (**A**) *C*_max_ and (**B**) AUC_0–24_ stratified by weight band. Boxplots represent the median and interquartile range (IQR). Whiskers are drawn to the furthest point within 1.5× the IQR. The dashed line (shaded region) represents the adult targets, *C*_max_ 35 mg/L (20–60) and AUC_0–24_ 363 mg*h/L (250–450) (20–24). *C*_max_, maximum concentration; AUC_0–24_, area under the 24-h concentration time curve.

The adequacy of WHO-recommended weight-based doses was explored in simulations (Fig. 3). Maximum concentration (*C*_max_) > 35 mg/L target attainment with WHO-recommended doses for the TB and TB/HIV groups was as follows: 4–<8 kg, 14.3 and 6.1%; 8–<12 kg, 45.7 and 34.7%; 12–<16 kg, 63.3 and 55%; 16–<25 kg, 56.7 and 60.6%; and 25–<35 kg, 60.7 and 60.6%. For area under the concentration time curve over 24 h (AUC_0–24_) >363 mg*h/L; 4–<8 kg, 5.4 and 0%; 8–<12 kg, 21 and 12.7%; 12–<16 kg, 41.8 and 24.8%; 16–<25 kg, 47 and 31.6%; and 25–<35 kg, 48.2 and 33.3%. Optimized doses are shown in Table 3. Compared to WHO-recommended doses averaging 35 mg/kg, optimized doses for both *C*_max_ and AUC_0-24_ averaged 39 mg/kg for TB and 42 mg/kg for TB/HIV. Target attainment across weight bands for *C*_max_ > 35 mg/L and AUC_0–24_ > 363 mg*h/L with the optimized doses ranged from 63 to 88% and 46 to 68% for the TB group and 76 to 91% and 47 to 65% for the TB/HIV group. Target attainment was lowest in the 4–<8 kg weight band, and simulations suggested the current recommended dose may need to be doubled.

**TABLE 3:** Optimized pyrazinamide doses for *C*_max_ and AUC_0–24_ target attainment at steady state by weight band in children with tuberculosis with or without HIV^*a*^

Weight band (kg)	WHO-recommended dose in mg (mg/kg)	Optimized dose (TB) in mg (mg/kg)	Optimized dose (TB/HIV) in mg (mg/kg)
*C*_max_	4 to <8	150 (19–38)	300 (38–75)	300 (38–75)
	8 to <12	300 (25–38)	375 (31–47)	450 (38–56)
	12 to <16	450 (28–38)	450 (28–38)	525 (33–44)
	16 to <25	600 (24–38)	675 (27–42)	675 (27–42)
	25 to <35	800 (23–32)	1,000 (29–40)	1,000 (29–40)
AUC_0–24_	4 to <8	150 (19–38)	300 (38–75)	300 (38–75)
	8 to <12	300 (25–38)	375 (31–47)	450 (38–56)
	12 to <16	450 (28–38)	525 (33–44)	525 (33–44)
	16 to <25	600 (24–38)	600 (24–38)	675 (27–42)
	25 to <35	800 (23–32)	800 (23–32)	1,000 (29–40)

^*a*^ *C*_max_, maximum concentration; AUC_0–24_, area under the 24-h concentration time curve; WHO, World Health Organization.

### Ethambutol

A total of 501 samples from 84 participants were included. One participant (TB/HIV) was removed from the analysis because all samples were BLQ or near BLQ, suggesting a missed dose or vomiting post-dose. Six additional samples were removed from various participants in the analysis because they appeared to have been labeled out of order. The median (range) dose administered was 21.4 mg/kg (14.3–34.2). EMB was best described as a two-compartment model with a tlag and first-order absorption and linear elimination (Table 2). Figure 1 and 2 show the goodness-of-fit plots and VPCs, respectively. Weight was allometrically scaled on Cl/F and V1/F using estimated coefficients (Cl/F: Cl/F*(Weight/15.1)^0.7 and V1/F: V1/F*(Weight/15.1)^0.62), resulting in an 82-point drop in OFV and improved diagnostic plots. Inclusion of HIV status in the model further resulted in a 17-point drop in OFV. Children with TB/HIV had 25% faster clearance than HIV-negative children. A combination of additive and proportional error models was utilized. Similar to PZA, incorporating maturation function, IOV on PK parameters, and HIV medications did not enhance the model. Notably, lopinavir (coadministered with ritonavir), which had a previous impact on EMB (25), was only used in four patients in the TB/HIV group.

**Fig 4:**
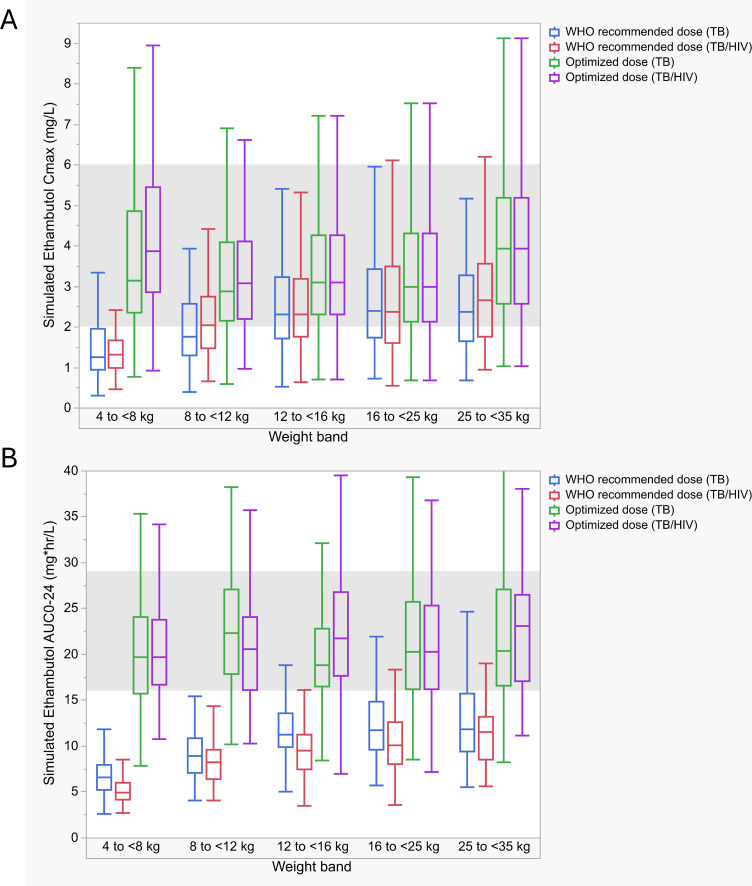
Simulated WHO-recommended and optimized ethambutol *C*_max_ and AUC_0–24_ values stratified by weight band and HIV status. Boxplots of simulated steady-state ethambutol (**A**) *C*_max_ and (**B**) AUC_0–24_ stratified by weight band. Boxplots represent the median and interquartile range. Whiskers are drawn to the furthest point within 1.5× the interquartile range. The shaded regions represent target *C*_max_ (2–6 mg/L) and AUC_0–24_ (16–29 mg*h/L), respectively (15, 20, 21, 23, 26). *C*_max_, maximum concentration; AUC_0-24_, area under the 24-h concentration time curve.

Simulations explored the adequacy of WHO-recommended weight-based doses (Fig. 4). *C*_max_ >2 mg/L attainment with WHO-recommended doses for the TB and TB/HIV groups was as follows: 4–<8 kg, 20.8 and 13.5%; 8–< 12 kg, 38.6 and 53.2%; 12–<16 kg, 64.6 and 61.5%; 16–<25 kg, 65.2 and 62.9%; and 25–<35 kg, 55.8 and 72.5%. For AUC_0–24_ > 16 mg*h/L; 4–<8 kg, 1.9 and 0%; 8–< 12 kg, 0.1 and 0.1%; 12–<16 kg, 13.1 and 3.7%; 16–<25 kg, 18.2 and 6.9%; and 25–<35 kg, 21.2 and 7.2%. Optimized doses are shown in Table 4. Compared with the average WHO-recommended dose of 20 mg/kg, TB-optimized doses averaged 33 mg/kg for *C*_max_ and 34 mg/kg for AUC_0–24_, while TB/HIV-optimized doses were higher, averaging 34 mg/kg for *C*_max_ and 50 mg/kg for AUC_0–24_. The highest mg/kg doses to optimize were in the 4–<8 and 8–<12 kg groups. Target attainment across weight bands for *C*_max_ > 2 mg/L and AUC_0–24_ > 16 mg*h/L with the optimized doses ranged from 79 to 85% and 61 to 83% for the TB group and 76 to 87% and 72 to 83% for the TB/HIV group.

**TABLE 4:** Optimized ethambutol doses for *C*_max_ and AUC_0–24_ target attainment at steady state by weight band in children with tuberculosis with or without HIV^*a*^

	Weight band (kg)	WHO-recommended dose in mg (mg/kg)	Optimized dose (TB) in mg (mg/kg)	Optimized dose (TB/HIV) in mg (mg/kg)
*C*_max_	4 to <8 kg	100 (13–25)	250 (31–63)	300 (38–75)
	8 to <12 kg	200 (17–25)	350 (29–44)	300 (24–38)
	12 to <16 kg	300 (19–25)	400 (25–33)	400 (25–33)
	16 to <25 kg	400 (16–25)	500 (20–31)	500 (20–31)
	25 to <35 kg	550 (16–22)	825 (24–33)	825 (24–33)
AUC_0–24_	4 to <8 kg	100 (13–25)	300 (38–75)	400 (50–75)
	8 to <12 kg	200 (17–25)	500 (42–63)	500 (42–63)
	12 to <16 kg	300 (19–25)	500 (31–42)	600 (38–50)
	16 to <25 kg	400 (16–25)	600 (24–38)	800 (32–50)
	25 to <35 kg	550 (16–22)	825 (24–33)	1,100 (31–44)

^*a*^ *C*_max_, maximum concentration; AUC_0–24_, area under the 24-hour concentration time curve; WHO, World Health Organization.

## DISCUSSION

We developed models to describe the population PK of children receiving EMB and PZA as part of first-line HRZE regimens for drug-susceptible TB. HIV status impacted PZA and EMB clearance. Applying allometric scaling was important to describe pediatric pharmacokinetics more precisely. Higher doses of PZA and EMB may warrant further evaluation when adult targets are used, with consideration for the risk of adverse effects.

PZA population PK was best described as a one-compartment model in congruence with previous models (16, 27–30). Covariates previously associated with impacting PZA PK include male sex, severe malnutrition, and HIV-positive status (20), but only HIV status improved model fit in our study. Clearance was about 18.5% faster in children with HIV/TB compared to those without HIV. Target attainment was modest, with less than half of the participants achieving adult *C*_max_ (>35 mg/L) and AUC_0–24_ targets (>363 mg*h/L) with WHO-recommended doses. This was most pronounced in the 4–<8 kg group, where the recommended dose needed to be increased from 150 to 300 mg in both the TB and TB/HIV groups to better optimize target attainment. Low PZA exposure has been consistently associated with lower culture conversion rates, treatment failure, recurrence, and death, and pediatric patients have had poor outcomes when PZA peak concentrations are <38.1 mg/L (20, 22, 31, 32). PZA target attainment has been previously identified as suboptimal (17, 18, 27). Kwara et al. examined whether the revised FDCs and dosing recommendations achieved the desired PK in 71 Ghanaian children with TB. Low PZA exposures were observed, and only 46% of participants achieved AUC_0–24_ above 339.9 mg*h/L (18). In contrast, Chabala and colleagues reported largely adequate PZA AUC_0–24_ target attainment in Zambian and South African children receiving updated FDCs and weight-band dosing, except among those weighing 4–<8 kg. However, they did use a range with a lower bound (233–429 mg*h/L) (19). While AUC_0–24_< 363 mg*h/L has been associated with poorer outcomes, PZA exposure targets remain incompletely defined, and multiple clinical factors contribute to treatment response (33). In our cohort, target attainment with optimized PZA doses was lower than the ~90% typically sought, reflecting the deliberate safety constraints applied during dose optimization. Achieving 90% attainment, especially for AUC-based targets, required concerningly high doses. Although increasing PZA doses might enhance target attainment, especially in lower weight children, the concentration-dependent risk of hepatotoxicity likely constrains the practicality of higher dosing (34, 35). Additionally, dosing feasibility with available FDCs is a consideration, as increasing the PZA dose would inevitably increase rifampin, isoniazid, and EMB exposure depending on the formulation.

EMB was best described using a two-compartment model similar to previously published models (25, 27). This is biologically plausible, as ethambutol elimination is biphasic, with the terminal elimination phase occurring as ethambutol is slowly released. Additionally, the apparent volume of distribution (V/F) in both compartments was large, potentially due to uptake in macrophages and erythrocyte binding (36–40). When compared to adult *C*_max_ (2–6 mg/L) or AUC_0–24_ (16–29 mg*h/L) ranges, simulated target attainment across weight bands was low, especially for the 4–<8 and 8–<12 kg groups. In these groups, achieving both *C*_max_ and AUC_0–24_ adult-equivalent exposures would require at least a twofold dose increase in HIV-negative children and an approximately threefold increase in those children with HIV. Similarly, Tikiso et al. proposed a doubling of dose would be needed for pediatric patients to achieve exposure within the 2–6 mg/L adult range or tripled in HIV+ children receiving lopinavir/ritonavir (25). Additionally, although a higher AUC_0–24_ target was used, Horita and colleagues found that among Ghanaian pediatric participants, no simulated EMB doses adequately achieved the target AUC_0–24_ of 23.6 mg*h/L across weight bands (27). EMB dose increases need to be balanced against toxicity concerns, particularly ocular toxicity, which can be difficult to monitor in young children. Additional research will be needed to further evaluate EMB dosing recommendations.

Simulated target attainment for both PZA and EMB was lowest in the 4–<8 kg group, a concern given the heightened vulnerability of young children to severe TB (41). Incomplete absorption may potentially be due to immature absorption processes. An additional contribution could be that weight-normalized clearance is higher in smaller individuals, who may need larger mg/kg doses to achieve similar concentrations as those with a larger body weight (42). Also, fixed-dosing options limit true weight-based dosing because patients at the higher end of the weight band receive a lower mg/kg dose, which can reduce target attainment to a degree. However, plasma concentrations alone cannot determine treatment outcomes. There are multiple other factors to consider, such as the susceptibility of TB bacilli and concentrations at the site of infection.

There are limitations to our study. First, this study only enrolled children at a single hospital in Ghana, which may limit its generalizability to other populations. Additionally, we could not examine the relationship between drug PK and outcomes, so we cannot comment on the association between anti-TB drug exposure and treatment success or failure. Finally, both the efficacy targets and the pragmatic safety constraints for EMB and PZA remain incompletely defined. The efficacy targets are based on historical data, and both thresholds for efficacy and safety should be interpreted as conservative estimates rather than definitive. Further research is needed to establish optimal dosing for EMB and PZA.

In conclusion, PZA was best characterized by a one-compartment model, while EMB followed a two-compartment model. Body weight played a crucial role in explaining interpatient variability, and HIV status influenced drug clearance. Optimized dosing to achieve adult-equivalent exposures required higher-than-current doses, particularly among children in the lowest weight bands and those with HIV. This raises concerns regarding risks for drug-associated toxicities and will require further evaluation.

## MATERIALS AND METHODS

### Study design

A two-arm PK study in children with TB with and without HIV coinfection was conducted at the Komfo Anokye Teaching Hospital in Kumasi, Ghana, as previously described (17). Briefly, children 3 months to 14 years old were enrolled from February 2019 to June 2021 and had PK sampling after at least 4 weeks of TB treatment with HRZE. Participants were dosed by weight band (PZA: 35 mg/kg [range 30–40 mg/kg]; EMB: 20 mg/kg [range 15–25 mg/kg]) (14, 39). For children weighing <25 kg, the new dispersible HRZ 50/75/150 mg plus single dispersible EMB (100 mg) tablets were used in the intensive phase and HR 50/75 mg tablets (MaCleods Pharmaceuticals, Mumbai, India) in the continuation phase. This corresponds to one, two, three, and four tablets of HRZ (50/75/150 mg) in the weight bands of 4–<8, 8–<12, 12–<16, and 16–<25 kg, respectively. For children ≥25 kg, the adult HRZE 75/150/400/275 mg tablets (Lupin, Ltd., Chikalthana, Aurangabad, India) were used in the intensive phase and HR 75/150 mg in the continuation phase. All medications were supplied through the Global TB Drug Facility. On the day of sampling, medication administration was directly observed by nurses following an overnight fast in non-breastfeeding children, while dosing prior to hospitalization for sampling was administered by family members at home. Parents and guardians provided informed consent, and signed assent was obtained from children older than 8 years. Institutional Review Board (IRB) approval was obtained from both Kwame Nkrumah University of Science and Technology (CHRPE/AP/590/18) and the University of Florida (IRB201801820).

Rich PK sampling was collected on one occasion from blood samples at 0 (pre-dose), 1-, 2-, 4-, 8-, and 12-h post-dose. Samples were processed, plasma aliquoted, and then stored at −80°C until shipment on dry ice to the University of Florida Infectious Disease Pharmacokinetics Laboratory. Drug concentrations were quantified using liquid chromatography tandem mass spectrometry (LC-MS/MS). The range of detection was 0.5–100 mg/L for PZA and 0.05–10 mg/L for EMB. The inter- and intra-batch accuracies and precision coefficient of variation were 93–102 and <11%, respectively. Concentrations above the range of quantification were diluted and reanalyzed. Samples with drug detected but BLQ were interval-censored in the models. BLQ samples with no drug detected are reported as 0 mg/L.

### Model building

Non-compartmental analysis (NCA) was performed prior to model building using Phoenix WinNonlin (Certara v8.3; Princeton, NJ, USA) to estimate the initial PK parameters. The results of NCA were published previously (17). Nonlinear mixed-effects models were used to describe the population PK using Monolix2024R1 (Lixoft, France) (43). The stochastic approximation expectation-maximization (SAEM) algorithm was used to estimate population parameters. The base structural models tested included one and two compartments. Absorption models tested included zero- and first-order with or without lag time (tlag), simultaneous zero- and first-order, sequential zero- and first-order, and transit compartments. Linear and nonlinear eliminations were tested. Structural models were assessed for fit based on OFV, Bayesian information criterion (BIC), goodness-of-fit plots, plausibility of estimated parameters, and relative standard errors (RSE). A lognormal distribution was assumed for the parameters. Standard errors were estimated from the Fisher information matrix using a stochastic approach, and additive, proportional, and combined error models were tested to evaluate residual variability.

Conditional sampling use for stepwise approach based on correlation tests (COSSAC) was used in covariate screening (44). A *P*-value of 0.05 was used for forward addition and 0.01 for backward deletion. Log continuous covariates tested included weight, fat-free mass (45), age, serum creatinine, eGFR (Schwartz equation), aspartate aminotransferase, alanine aminotransferase, and total bilirubin. Categorical covariates tested included sex, HIV status, HIV medications, dose formulation (child or adult), and malnutrition (<−2 standard deviations body mass index-for-age Z score). The reduction in between-subject variability in PK parameters and improvement in diagnostic plots were evaluated before retaining covariates. Allometric scaling using estimated and previously reported values (Cl/F*(weight/reference weight)^0.75 and V/F*(weight/reference weight)^1) was tested to improve model fit (46). Additionally, a maturation function (46) was tested on clearance, and the impact of IOV was evaluated on PK parameters. VPCs evaluated the predictive performance of the final model using 1,000 simulated data sets. The fifth, 50th, and 95th percentiles were then compared against the observed concentrations.

The final models were used to simulate steady-state *C*_max_ and AUC_0–24_ in Simulx2024R1 (Lixoft, France) (47) stratified by WHO-recommended weight band dosing (4–<8, 8–<12, 12–<16, 16–<25, and 25–<35 kg). To more accurately represent the ≥25 kg group, it was redefined as 25–<35 kg, as only two patients exceeded 35 kg. Patient demographics in the simulation mirrored those in the final models, and characteristics based upon weight bands were replicated 1,000 times. The dosing frequency was once daily. The following available doses were considered for simulations and split as needed: 150 and 400 mg for PZA and 100 mg and 275 mg for EMB. Concentrations were simulated in increments of 0.5 h. Simulated steady-state PZA exposures were evaluated against adult targets (*C*_max_: 35 mg/L and AUC_0–24_: 363 mg*h/L) and contextualized using broader exposure ranges (*C*_max_: 20–60 mg/L and AUC_0–24_: 250–450). For EMB, exposures were compared with adult *C*_max_ (2–6 mg/L) and AUC_0–24_ (16–29 mg*h/L), with the lower bound considered the target (12, 20–24, 26, 48, 49). Because formal exposure-toxicity relationships for ethambutol and pyrazinamide are not well established in children, optimized doses were selected to maximize target attainment while ideally maintaining no more than 15% (max 20%) of simulated patients above the typical range. This approach provides a conservative safety constraint without assuming a specific exposure-toxicity model.

### Statistical analysis

Statistics were performed and figures generated using JMP Pro v18 (Cary, NC, USA). Continuous data are presented using median (range) and categorical data as count (percentage). The Wilcoxon rank sum test compared continuous variables, and categorical variables were compared using the chi square (*χ*^2^) test or Fisher’s exact test for small counts.
